# Knowledge, Preference, and Adverse Effects of Xylazine Among Adults in Substance Use Treatment

**DOI:** 10.1001/jamanetworkopen.2024.0572

**Published:** 2024-02-28

**Authors:** Martin Hochheimer, Justin C. Strickland, Jill A. Rabinowitz, Jennifer D. Ellis, Kelly E. Dunn, Andrew S. Huhn

**Affiliations:** 1 Department of Psychiatry and Behavioral Sciences, Johns Hopkins University School of Medicine, Baltimore, Maryland; 2 Department of Psychiatry, Robert Wood Johnson Medical School, Rutgers University, Piscataway, New Jersey

## Abstract

This cross-sectional study evaluates aspects of xylazine adulteration of opioids among individuals entering substance use disorder treatment.

## Introduction

Xylazine, an α_2_-agonist, is a veterinary tranquilizer that is increasingly found in nonmedical opioids in the US and is associated with increased risk for skin ulcerations and complications associated with opioid overdose reversal with naloxone (as a nonopioid sedative)^1^ and opioid withdrawal management.^2^ Qualitative research suggests the sedative effect of xylazine may be preferred by some due to its perceived ability to prolong the effect of fentanyl.^3^ However, knowledge is limited regarding public awareness, preference, adverse effects, and changes in the opioid withdrawal syndrome with xylazine exposure. This cross-sectional study evaluated these aspects of xylazine adulteration of opioids among patients entering substance use disorder treatment.

## Methods

This cross-sectional study followed the Strengthening the Reporting of Observational Studies in Epidemiology (STROBE) reporting guideline. Informed consent and review were waived because it was recognized as nonhuman participant research by the Johns Hopkins School of Medicine institutional review board.

Individuals in 78 substance use treatment programs in the US, who reported nonmedical opioid use within 30 days prior to treatment were surveyed about xylazine adulteration from January to October 2023. Trac9, a commercial treatment outcomes tool, administered the assessments and shared the results via a data use agreement.

Respondents answered questions about demographics (including race and gender to assess for potential disparities) and xylazine awareness. The participants were asked to self-report race or ethnicity from a list which included the following options: African American, Asian, Hispanic or Latino, Native American, Native Hawaiian or Pacific Islander, White, or other. Those who were aware of xylazine were also asked about the perceived likelihood of past xylazine adulteration of opioids, lifetime exposure to xylazine, and desire and preference for xylazine. All respondents were asked about developing specific adverse effects or experiencing withdrawal symptoms. The complete survey can be found in the eAppendix in Supplement 1. χ^2^ tests using Fisher exact post hoc analysis evaluated whether there were demographic differences in xylazine awareness, desire for xylazine, and preference for xylazine. Logistic regression was used to examine the association between the perceived likelihood of xylazine adulteration and the presence or absence of specific adverse effects and withdrawal effects; significance was set at α <.05, with Holm-Bonferroni correction used for post hoc tests.^4^ R version 4.3.2 (R Project for Statistical Computing) was used for analyses.

## Results

There were 2872 respondents (1970 [69%] male; 895 [31%] female; 294 Black or African American respondents [10%]; 283 Hispanic or Latino respondents [10%]; 2127 White respondents [74%]). Of these, 1304 respondents (45%) were aware of xylazine, with significantly higher awareness among those who reported opioids as their primary substance (550 respondents [49%]; *P* <.001). Overall, 1166 respondents (90%) who were aware of xylazine did not want xylazine in their opioids, and 932 respondents (85%) did not prefer opioids containing xylazine. Non-Hispanic White individuals (1023 respondents [48%]; *P* < .001) and those who identified heroin or fentanyl (485 respondents [52%]; *P* < .001) or commercial opioids (550 respondents [49%]; *P* = .002) as their primary substance were most likely to have knowledge of xylazine adulteration compared with people who reported a different primary substance. Conversely, Hispanic or Latino individuals (99 respondents [35%]; *P* < .001) and those who primarily used alcohol (59 respondents [28%]; *P* < .001) or methamphetamine (100 respondents [31%]; *P* < .001) were significantly less likely to have awareness of xylazine. No demographic differences emerged concerning the desire or preference for xylazine (Table 1).

**Table 1.** Xylazine Knowledge and Preference With Patient Demographics

Variable	No. (%) (N = 2872)^a^	Have you heard of xylazine? (n = 2872)			Do you want xylazine in the opioids you use? (n = 1283)^b^			Do you prefer xylazine in the opioids you use? (n = 1100)^b^		
		Yes	No	*P* value^c^	Yes	No	*P* value^c^	Yes	No	*P* value^c^
Primary substance^d^										
Alcohol	208 (7)	59 (28)	149 (72)	<.001	5 (7)	63 (93)	.67	9 (16)	48 (84)	>.99
Benzodiazepine	47 (2)	20 (43)	27 (57)	.77	1 (7)	14 (93)	1.00	2 (20)	8 (80)	>.99
Cannabis	32 (1)	6 (19)	26 (81)	<.001	0	8 (100)	.63	1 (17)	5 (83)	>.99
Cocaine	187 (7)	75 (40)	112 (60)	.15	4 (6)	66 (94)	.40	4 (8)	49 (92)	.12
Heroin or fentanyl	938 (33)	485 (52)	453 (48)	<.001	50 (11)	422 (89)	.19	77 (18)	351 (82)	.05
Methamphetamine	318 (11)	100 (31)	218 (69)	<.001	5 (5)	102 (95)	.11	7 (8)	79 (92)	.06
Opioids	1120 (39)	550 (49)	570 (51)	<.001	50 (9)	483 (91)	.84	65 (14)	387 (86)	.50
Stimulants	22 (1)	9 (41)	13 (59)	.83	2 (20)	8 (80)	.23	3 (38)	5 (62)	.11
Race or ethnicity^e^										
African American	294 (10)	117 (40)	177 (60)	.05	17 (15)	100 (85)	.04	15 (15)	85 (85)	>.99
Asian	13 (0.5)	4 (31)	9 (69)	.40	0	0	NA	0	3 (100)	>.99
Hispanic or Latino	283 (10)	99 (35)	184 (65)	<.001	4 (4)	91 (96)	.10	9 (11)	71 (89)	.34
Native American	54 (2)	21 (39)	33 (61)	.34	1 (4)	22 (96)	.51	2 (11)	17 (89)	.75
Native Hawaiian or Pacific Islander	14 (0.5)	4 (29)	10 (71)	.28	1 (20)	4 (80)	.38	1 (17)	5 (83)	>.99
Other^f^	87 (3)	36 (41)	51 (59)	.45	2 (5)	36 (95)	.57	6 (20)	24 (80)	.61
White	2127 (74)	1023 (48)	1104 (52)	<.001	92 (9)	909 (91)	1.00	135 (16)	727 (84)	.54
Sex^g^										
Female	895 (31)	397 (44)	498 (56)	.47	41 (10)	353 (90)	.29	57 (18)	260 (82)	.12
Male	1970 (69)	906 (46)	1064 (54)	.35	76 (9)	811 (91)	.34	110 (14)	671 (86)	.10
Other	7 (0.2)	1 (14)	6 (86)	.14	0	2 (100)	1.00	1 (50)	1 (50)	.28
Employed^h^										
Yes	754 (26)	339 (45)	415 ((55)	.80	20 (6)	315 (94)	.02	26 (10)	247 (90)	.002
No	2112 (74)	962 (46)	1150 (54)	.80	97 (10)	848 (90)	.02	142 (17)	683 (83)	.002
Type of care^i^										
Supervised withdrawal	1174 (41)	540 (46)	634 (54)	.62	58 (11)	465 (89)	.05	76 (16)	396 (84)	.55
Intensive outpatient	219 (8)	114 (52)	105 (48)	.04	3 (3)	110 (97)	.02	5 (6)	83 (94)	.01
Residential	1479 (51)	650 (44)	829 (65)	.11	56 (9)	591 (91)	.56	87 (16)	453 (84)	.45

Abbreviation: NA, not available.

^a^ No. and percentage of individuals in each of the demographic categories. For all other cells, the No. (%) compares the respondents who answered yes and no; the numbers may not add up to 100% due to rounding.

^b^ Only those who endorsed having heard about xylazine were asked this question.

^c^ *P* values in line with categories are for omnibus χ^2^ test, those in line with responses are for the Fisher exact post hoc analysis.

^d^ The overall *P* value for knowledge of xylazine was <.001; for use of xylazine, .39; for preference for xylazine, .11.

^e^ The overall *P* value for knowledge of xylazine was <.001; for use of xylazine, .16; for preference for xylazine, .87.

^f^ Consists of those who chose to identify as other rather than any other category provided.

^g^ The overall *P* value for knowledge of xylazine was .18; for use of xylazine, .52; for preference for xylazine, .10.

^h^ The overall *P* value for knowledge of xylazine was .81; for use of xylazine, .02; for preference for xylazine, .003.

^i^ The overall *P* value for knowledge of xylazine was .07; for use of xylazine, .02; for preference for xylazine, .03.

Overall, 1963 respondents (68%) reported adverse effects and 1163 respondents (41%) reported xylazine-associated altered withdrawal symptoms. Skin lesions, increased sedation, loss of consciousness, and difficulty in reversing overdoses with naloxone were more common among persons who believed their opioids may have contained xylazine and/or preferred xylazine (Table 2). However, only 2 of the 4 potential withdrawal effects—increased headaches during withdrawal and a sensation of burning (particularly when given naloxone)—were positively associated with past 30-day xylazine exposure.

**Table 2.** Results of Logistic Regressions Evaluating the Association Between the Estimator Questions and the Outcome Measures

Outcome measures	Questions used as estimators								
	How likely is it that you were given xylazine with your opioids during the last 30 d?^a^			How likely is it that you were ever given xylazine with your opioid?^b^			Do you prefer using xylazine with your opioids?^c^		
	No.^d^	OR (95% CI)	*P* value	No.^d^	OR (95% CI)	*P* value	No.^d^	OR (95% CI)	*P* value
Adverse effects									
Skin lesions	798	1.55 (1.38-1.75)	<.001	1302	1.16 (1.11-1.21)	<.001	940	1.82 (1.22-2.67)	.003
Opioids more sedating	949	1.63 (1.48-1.79)	<.001	1586	1.28 (1.23-1.32)	<.001	1100	2.07 (1.48-2.93)	<.001
Fallen asleep after using opioids	949	1.46 (1.33-1.62)	<.001	1586	1.18 (1.15-1.22)	<.001	1100	1.50 (1.06-2.16)	.03
Had an overdose that was difficult to reverse with naloxone	949	1.18 (1.06-1.30)	.002	1586	1.08 (1.04-1.12)	<.001	1100	1.49 (1.03-2.12)	.03
Withdrawal effects									
High blood pressure	571	1.06 (0.94-1.20)	.35	959	1.07 (1.05-1.11)	.005	671	1.37 (0.91-2.03)	.13
Headaches	571	1.19 (1.06-1.35)	.004	959	1.09 (1.05-1.14)	<.001	671	1.50 (1.00-2.28)	.05
Feeling of electricity	571	1.06 (0.93-1.20)	.39	959	1.05 (1.01-1.10)	.023	671	1.27 (0.84-1.92)	.25
Feeling that body is on fire (especially when naloxone is administered)	571	1.18 (1.04-1.35)	.009	959	1.10 (1.05-1.15)	<.001	671	1.59 (1.06-2.37)	.02

Abbreviation: OR, odds ratio.

^a^ Scale 0 to 5.

^b^ Scale 0 to 10.

^c^ Dichotomous.

^d^ Number of respondents to both variables.

## Discussion

This study provides insight into xylazine’s presence in the opioid market based on perceptions of individuals in substance use disorder treatment. Findings indicate a widespread lack of awareness among persons using opioids regarding risk for xylazine exposure and potential inclusion of xylazine within their opioid supply. Preference for xylazine was associated with increased adverse effects. A limitation of this study is that the sample consisted entirely of individuals within treatment programs, potentially limiting generalizability to others who use opioids. The lack of awareness of xylazine, coupled with reported hazardous adverse effects and altered withdrawal symptoms, emphasizes the urgent necessity for harm reduction initiatives to increase public awareness of xylazine and help mitigate xylazine exposure and associated consequences.^5^

## Notes

Supplement 1. **eAppendix.** Survey

Supplement 2.
Data Sharing Statement
