# Supplementary material for: Knowledge, Preference, and Adverse Effects of Xylazine Among Adults in Substance Use Treatment
Source: JAMA Netw Open. 2024 Feb 28;7(2):e240572. doi: 10.1001/jamanetworkopen.2024.0572 (PMC10902730; doi:10.1001/jamanetworkopen.2024.0572)
Supplement: Supplement 1. — eAppendix. Survey [file jamanetwopen-e240572-s001.pdf]

Supplemental Online Content

Hochheimer M, Strickland JC, Rabinowitz JA, Ellis JD, Dunn KE, Huhn AS. Knowledge, preference, and perceived association of xylazine among adults in substance use treatment. *JAMA Netw Open*. 2024;7(2):e240572.
doi:10.1001/jamanetworkopen.2024.0572

eAppendix 1. Survey

This supplemental material has been provided by the authors to give readers additional information about their work.

© 2024 Hochheimer M et al. *JAMA Network Open.*

eAppendix

Survey

Introduction – which is read or displayed on screen prior to survey administration.

Xylazine is a sedative that is commonly used in veterinary medicine. In the past year, there have been reports of xylazine is being mixed with fentanyl or heroin in some areas. This is often called tranq/fent or tranq/dope, or blackout, though it may have other names as well. We are asking to understand your thoughts and experiences with xylazine.

1. Before this message, have you heard of xylazine or tranq?
    a. Yes/no/unsure - the answer is no then skip to question 6
2. How concerned are you about being exposed to xylazine?
    a. 0-(not at all) – 10 (extremely)
3. How likely do you think that xylaxine/ tranq **was ever in your lifetime** mixed into the heroin/fentanyl you use?
    a. 0 (not likely) – 10 (almost certain) – or don't know
4. How often in **the last 30 days** do you believe that the heroin/fentanyl you used had xylazine/tranq mixed in?
    a. None of the time
    b. Hardly any of the time
    c. A little of the time
    d. Some of the time
    e. A good bit of the time
    f. Most of the time
    g. All of the time
5. Do you want xylazine to be mixed into your heroin/fentanyl?
    a. Yes/no/ unsure
6. In the past year have you experienced a heroin/fentanyl withdrawal syndrome that felt much different from other post withdrawals syndromes?
    a. Yes/no/unsure
7. If yes to question 6
    a. How was it different? Open ended ask for all symptoms then check for the following specific
    b. Did you experience?
        i. High blood pressure –
            1. yes/no/don't know or unsure
        ii. Headaches
            1. yes/no/don't know or unsure
        iii. Feeling of electricity
            1. yes/know/ don't know or unsure
        iv. Feeling like your body is on fire (especially when given Narcan)
            1. yes/no/don't know or unsure/ never given Narcan

© 2024 Hochheimer M et al. *JAMA Network Open.*

8. In the past year have you developed skin lesions or sores that do not heal, related to injection drug use?
    a. Yes/ no / unsure/ I don't inject
9. In the past year have you felt that your heroin/fentanyl was more sedating than normal?
    a. Yes/no/unsure
10. In the past year have you fallen asleep right after use or blacked out after using heroin/fentanyl?
    a. Yes/No/ Unsure
11. In the past year have you had an overdose that Narcan had trouble reversing?
    a. Yes/no/unsure/I was never given Narcan

© 2024 Hochheimer M et al. *JAMA Network Open.*
