# Supplementary material for: Knowledge, Preference, and Adverse Effects of Xylazine Among Adults in Substance Use Treatment
Source: JAMA Netw Open. 2024 Feb 28;7(2):e240572. doi: 10.1001/jamanetworkopen.2024.0572 (PMC10902730; doi:10.1001/jamanetworkopen.2024.0572)
Supplement: Supplement 2. — Data Sharing Statement [file jamanetwopen-e240572-s002.pdf]

Data Sharing Statement

Hochheimer. Knowledge, Preference, and Adverse Effects of Xylazine Among Adults in Substance Use Treatment. *JAMA Netw Open*. Published February 28, 2024. doi:10.1001/jamanetworkopen.2024.0572

Data
Data available: No

Additional Information
Explanation for why data not available: The data is proprietary to a third party as described in the manuscript.
